# The 2 × 2 Standpoints Model of Achievement Goals

**DOI:** 10.3389/fpsyg.2016.00742

**Published:** 2016-05-19

**Authors:** Rachel M. Korn, Andrew J. Elliot

**Affiliations:** Clinical and Social Sciences in Psychology, University of RochesterRochester, NY, USA

**Keywords:** achievement goal, standpoints, standards, development, demonstration

## Abstract

In the present research, we proposed and tested a 2 × 2 standpoints model of achievement goals grounded in the development-demonstration and approach-avoidance distinctions. Three empirical studies are presented. Study 1 provided evidence supporting the structure and psychometric properties of a newly developed measure of the goals of the 2 × 2 standpoints model. Study 2 documented the predictive utility of these goal constructs for intrinsic motivation: development-approach and development-avoidance goals were positive predictors, and demonstration-avoidance goals were a negative predictor of intrinsic motivation. Study 3 documented the predictive utility of these goal constructs for performance attainment: Demonstration-approach goals were a positive predictor and demonstration-avoidance goals were a negative predictor of exam performance. The conceptual and empirical contributions of the present research were discussed within the broader context of existing achievement goal theory and research.

## Introduction

The achievement goal approach to achievement motivation is over 40 years old and has generated a voluminous and varied body of conceptual and empirical work. It can be difficult for a literature of this size and scope to maintain coherence, as inevitably different perspectives and positions emerge on how to define, operationalize, and summarize research on models and the constructs within them. In this article, we offer an organizational structure for the achievement goal literature that is firmly grounded in the earliest theorizing on achievement goals (Maehr and Nicholls, 1980; Dweck and Elliott, 1983; Nicholls, 1984), and is fully compatible with contemporary theorizing as well. We provide specific labels—standpoints and standards—and specific terminology—development-demonstration and task/self-other—to help clarify the different models of achievement goals that have been proffered over the years. Doing so enables us to identify a surprisingly overlooked achievement goal model, the 2 × 2 standpoints model. We proceed to place this 2 × 2 standpoints model within the historical context of the achievement goal literature, and then present three studies designed to empirically test the structure and predictive utility of this model.

### Achievement goal models

In the initial, dichotomous model of achievement goals proposed in the 1980s, scholars differentiated between two types of goals that varied accordingly to the *focus of competence:* mastery goals (also called task goals), in which the focus was on developing competence and acquiring task mastery, and performance goals (also called ego goals), in which the focus was on demonstrating competence and outperforming others (Nicholls, 1984; Dweck, 1986; see Ames, 1992 on terminology). Although, not explicitly acknowledged at the time, the goals in this initial model were actually comprised of two distinct subcomponents, each of which could be considered separately (Elliot, 1999; Urdan, 2000). One subcomponent distinguished between different *standpoints on competence*—that is, viewing competence from the standpoint of developing it vs. demonstrating it; the other subcomponent distinguished between different *standards of competence*—that is, evaluating competence with regard to task/self-based vs. other-based standards. Thus, mastery goals represented a focus on both developing competence and using a task/self-based standard of competence evaluation, and performance goals represented a focus on both demonstrating competence and using an other-based standard of competence evaluation^1^.

**Figure 1:**
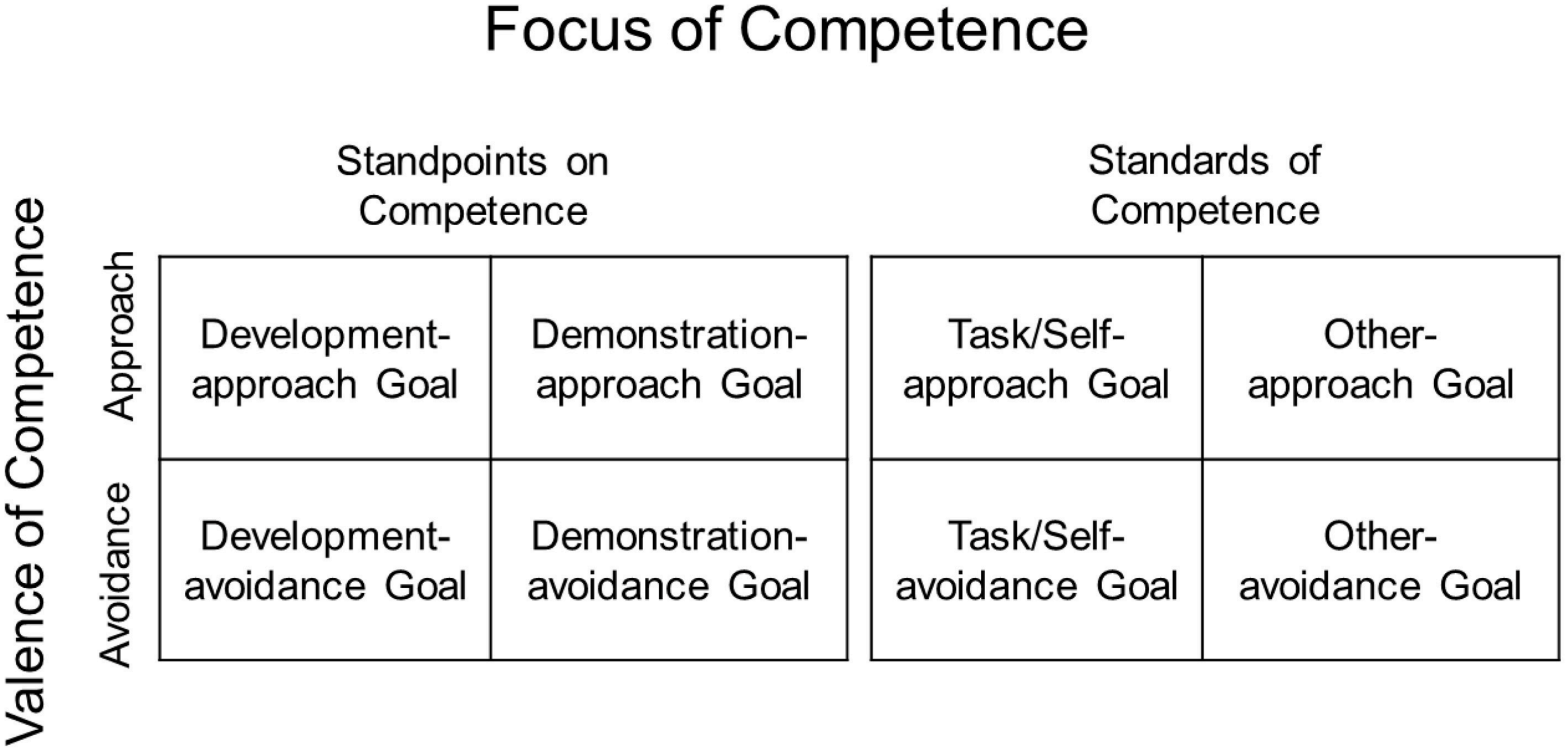
**Overview of the components and subcomponents of competence in achievement goal models**.

In the 1990s, achievement goal theorists began to include an additional component of competence in their conceptual work, beyond the focus of competence component. This additional component—the *valence of competence*—distinguishes between goals focused on approaching success and goals focused on avoiding failure (Elliot and Harackiewicz, 1996; see Figure 1 for an overview of the components and subcomponents of competence discussed herein). The valence of competence component has roots in classic theorizing on achievement motivation (Lewin et al., 1944; McClelland et al., 1953), and is an integral part of current achievement goal theory and research (Elliot, 1999). This revised conceptualization of achievement goals kept mastery goals intact but bifurcated performance-based goals by approach-avoidance, resulting in a three goal trichotomy: mastery, performance-approach, and performance-avoidance. Conceptually and operationally (i.e., in achievement goal measures), these goals retained both the standpoints on competence (development vs. demonstration) and the standards of competence (task/self vs. other) subcomponents (Elliot and Church, 1997; Middleton and Midgley, 1997; Skaalvik, 1997; Vandewalle, 1997). Thus, mastery goals focused on developing task mastery, whereas performance-approach goals focused on the demonstration of competence relative to others and performance-avoidance goals focused on avoiding the demonstration of incompetence relative to others.

A few years later, a 2 × 2 model was proposed in which both mastery and performance were fully crossed with approach and avoidance (Elliot, 1999; Pintrich, 2000). This model was accompanied by a shift to just one of the two subcomponents of the focus of competence—the standards of competence subcomponent (task/self vs. other). Thus, mastery-based goals focused on attaining success or avoiding failure relative to the absolute demands of the task or one's own past performance, whereas performance-based goals focused on attaining success or avoiding failure relative to others. This shift at the conceptual level was matched operationally in some achievement goal measures that focused exclusively on the standards of competence (task/self vs. other; Van Yperen, 2006; Elliot and Murayama, 2008; Riou et al., 2012). Other measures, however, continued to include items assessing the standpoints on competence (development vs. demonstration) along with the standards of competence (particularly with regard to performance-based goals; Hulleman et al., 2010 see for a review).

Subsequent research led to the bifurcation of mastery goals in terms of task-based and self-based standards (Elliot et al., 2011). Conceptually, this model focused exclusively on the standards of competence and did not include the standpoints on competence. The model identified three different standards to evaluate competence: the absolute demands of a task (task), one's own performance trajectory (self), and the performance of others (other); these standards of competence were fully crossed with approach and avoidance to produce a 3 × 2 achievement goal model and a corresponding standard-focused achievement goal questionnaire (Elliot et al., 2011; Wu, 2012; Johnson and Kestler, 2013; Mascret et al., 2015).

In sum, conceptually, achievement goal models have articulated the focus of competence two different ways: in terms of standpoints on competence (development vs. demonstration) and in terms of standards of competence (task/self vs. other); some models have collapsed these two subcomponents together, whereas others have focused exclusively on the standards of competence subcomponent. Operationally, we see the same: some measures of achievement goals have collapsed the two subcomponents together, whereas others have focused exclusively on the standards of competence. Importantly, both subcomponents of the focus of competence—standpoints and standards—may be considered equally central to the conceptualization of achievement goals. Furthermore, the valence of competence—approach vs. avoidance—is as applicable to the standpoints subcomponent as it is to the standards subcomponent. Nevertheless, researchers have yet to propose and test a model that focuses specifically on the standpoints subcomponent (development vs. demonstration) and fully crosses it with the valence component (approach vs. avoidance). This is surprising, given that the standpoints subcomponent has been characterized by some as the essence or core of the achievement goal (or at least the performance goal) construct, with the standards subcomponent described as a non-essential aspect (Elliott and Dweck, 1988; Grant and Dweck, 2003).

### The 2 × 2 standpoints model

The closest approximation to the 2 (standpoints) × 2 (valence) standpoints model has been offered in the social domain by Ryan and Shim (2006, 2008; see also a conceptual note by Elliot et al., 2011). The goals in their framework are conceptualized as different orientations toward social competence that guide individuals' behavior in social situations and beyond. Three types of goals are posited: social development(-approach) goals focused on “developing social competence,” social demonstration-approach goals focused on “demonstrating social competence and gaining from others positive judgments that one is socially desirable,” and social demonstration-avoid goals focused on “demonstrating that one does not lack social competence” (Ryan and Shim, 2006, p. 1247). These goals do not include the standards of competence subcomponent; instead, they focus solely on the development-demonstration distinction regarding social competence. Ryan and Shim (2006) created a questionnaire to assess their proposed goal constructs, and research has supported the hypothesized trichotomous model by linking the three goals to different outcomes (e.g., prosocial behavior, aggression, help-seeking behavior, psychological well-being; Horst et al., 2007; Ryan and Shim, 2008; Mouratidis and Sideridis, 2009; Kuroda and Sakurai, 2011; Ryan and Shin, 2011; Shim and Ryan, 2012; Rodkin et al., 2013; Shim et al., 2013a,b).

Within the academic domain, a few other researchers have focused specifically on demonstration reasons or goals and crossed them with valence. Elliot (1999) and Urdan (2000) noted that performance-based goals contained two competence aspects that could be separated (demonstration and a normative standard), and Grant and Dweck (2003) measured “ability” (akin to demonstration-approach) goals and established their predictive utility with regard to several outcomes (e.g., intrinsic motivation, effort expenditure). Urdan and Mestas (2006) proposed that students have different reasons for pursuing performance-based goals, including appearance-approach (akin to demonstration-approach) and appearance-avoidance (akin to demonstration-avoidance) reasons; they interviewed students, and categorized their free-responses using this framework (although operationally, some of the free-responses included both demonstration-based reasons and normative standards). In a meta-analysis, Hulleman et al. (2010) coded existing performance-approach goal measures according to their appearance-approach vs. appearance-avoidance focus, and showed the predictive utility of this bifurcation. Warburton and Spray (2014) measured appearance-approach and appearance-avoidance goals and demonstrated their differential links with effort and performance in the physical education domain.

Thus, theory and research on achievement goals has attended to portions of the full 2 × 2 standpoints model, focusing on three of the four goals in the social domain and two of the four goals in the academic domain. The full crossing of the development vs. demonstration and approach vs. avoidance distinctions has yet to be considered (in any domain); this is what we do in the present research. Specifically, the present research is comprised of three studies that propose and test a 2 (standpoints on competence: development vs. demonstration) × 2 (valence of competence: approach vs. avoidance) achievement goal model—herein labeled the 2 × 2 standpoints model.

### The present research

In Study 1 of the present research we created items (the 2 × 2 Development-Demonstration Achievement Goal Questionnaire; DAGQ) that assessed each of the four goals in the proposed model, collected data on the items, and examined the fit of the data to the hypothesized model. We predicted separation of the goal items by both the development vs. demonstration and the approach vs. avoidance distinctions.

In Study 2 we examined the links between these goals and intrinsic motivation. Conceptually, the development aspect of goals is likely to promote intrinsic motivation because it facilitates an internally-focused, process-oriented commitment to the task that supports full effort expenditure and persistence, whereas the demonstration aspect of goals is likely to undermine intrinsic motivation because it prompts other-focused, outcome-oriented striving that encourages strategic self-presentation and self-protective regulation (Nicholls, 1989; Dweck, 2000; Edwards, 2014; Senko and Tropiano, in press). The approach aspect of goals is likely to promote intrinsic motivation because it facilitates challenge appraisals and task absorption, whereas the avoidance aspect of goals is likely to undermine intrinsic motivation because it evokes threat appraisals, anxiety, and self-concern (Elliot, 2005; Van Yperen, 2006; Hulleman et al., 2010; Senko and Tropiano, in press). Thus, we expected that development-approach goals would be a positive predictor of intrinsic motivation, and demonstration-avoidance goals would be a negative predictor of intrinsic motivation. The patterns for demonstration-approach and development-avoidance goals are more difficult to anticipate, as in each case, the two aspects of each goal are expected to have different influences on intrinsic motivation (e.g., the development aspect of development-avoidance goals would be expected to facilitate intrinsic motivation, but the avoidance aspect would be expected to undermine it). As such, specific predictions are not offered for these goals. We controlled for social desirability in a subset of our analyses to ensure that any observed results were not merely a function of this potential “third variable.”

In Study 3 we examined the links between the 2 × 2 goals and performance attainment. Conceptually, the development aspect of goals is likely to facilitate deep learning, effort expenditure, and persistence over time that should facilitate long-term performance and retention, but may not benefit short term performance, especially on tasks simply requiring rote memorization (Dweck, 1986; Harackiewicz et al., 2002; Kaplan and Maehr, 2007). The demonstration aspect of goals is likely to prompt outcome oriented striving fueled by a desire for recognition or validation that could bolster short-term performance, but the accompanying impression management and self-worth concerns may be so distracting that they erode performance in the long-run, and possibly even the short run (Grant and Dweck, 2003; Urdan and Mestas, 2006; Hulleman et al., 2010; Warburton and Spray, 2014). The approach aspect of goals is likely to promote performance in the short- and long-run, because it promotes full task engagement, effort expenditure, and persistence, whereas the avoidance aspect of goals is likely to undermine performance in the short- and long-run because it evokes worry, task distraction, and self-handicapping processes (Elliot, 1999; Baranik et al., 2010; Burnette et al., 2013; Van Yperen et al., 2014). Our assessment of performance in Study 3 focused on short-term, normatively graded performance on a task primarily requiring rote memorization (but also some depth of understanding). Thus, our strongest expectation was that demonstration-avoidance goals would be negative predictors of exam performance; development-approach goals were not expected to emerge as clear (positive or negative) predictors of exam performance, given that the nature of the task and evaluative context was not optimal for these goals to facilitate performance (for analogous reasoning, see Harackiewicz et al., 2002; Midgley et al., 2002). Similar to Study 2, the patterns for demonstration-approach and development-avoidance goals are difficult to anticipate, as in each case, the two components of each goal are expected to have different or mixed influences on performance. As such, specific predictions are not offered for these goals. We controlled for Scholastic Aptitude Test (SAT) scores in a subset of our analyses to ensure that any observed results were not merely a function of this potential “third variable.”

Intrinsic motivation and performance attainment are arguably the two gold standard outcomes in research on achievement motivation, as both are of clear importance and one (intrinsic motivation) assesses the quality of engagement while the other (performance attainment) assesses the quantity of knowledge acquisition. Thus, linking the focal goals to these outcomes would represent strong validation of the 2 × 2 standpoints model.

## Study 1

### Method

#### Participants and procedure

A total of 244 individuals participated for modest monetary compensation (0.10 USD). Demographic information was not collected in this study. Participants followed a web link through Amazon's Mechanical Turk to access the study. Participation was restricted to individuals in the U.S. with an approval rating of 95% or higher. No manipulations and no data exclusions were used in any of the studies in the current research; in each study, all variables that were analyzed for this research are reported. Sample sizes were set a priori [a minimum of 200 participants for Study 1 and a minimum of 400 participants for Study 2 (the studies were stopped as soon as we became aware that the minimum threshold had been exceeded), and the maximum number of volunteers in the target course for Study 3]. All studies were approved by the university's research subjects review board before data collection began, and all procedures conformed to the relevant regulatory standards.

A welcome screen communicated that the study focused on individuals' achievement goals for an anagram task. Participants were informed that they would be answering a series of questions about their achievement goals prior to completing an anagram task. A detailed set of instructions for the achievement goal questionnaire were then provided. These instructions clearly defined the concept of a goal, and encouraged participants to read each item carefully and respond thoughtfully. After participants completed the questionnaire, they were told that they did not need to complete the anagram task. They were thanked for their participation and informed that the study was over.

#### Measures

In creating the questionnaire, we crafted a pool of candidate items for each of the focal achievement goals. These candidate items were considered with an eye toward selecting a small set of face-valid items to represent each goal. Twelve items, three for each of the four goal constructs, were selected for inclusion in the DAGQ: development-approach (e.g., “My goal is ‘To increase competence.”’), development-avoidance (e.g., “My goal is ‘To avoid a decrease in ability.”’), demonstration-approach (e.g., “My goal is ‘To demonstrate ability.”’), and demonstration-avoidance (e.g., “My goal is ‘To avoid demonstrating that I lack knowledge.”’). The full set of items may be seen in Appendix A of Supplementary Material). Participants responded to the items on a 1 (*not at all true of me*) to 5 (*extremely true of me*) scale.

### Results

**Figure 2:**
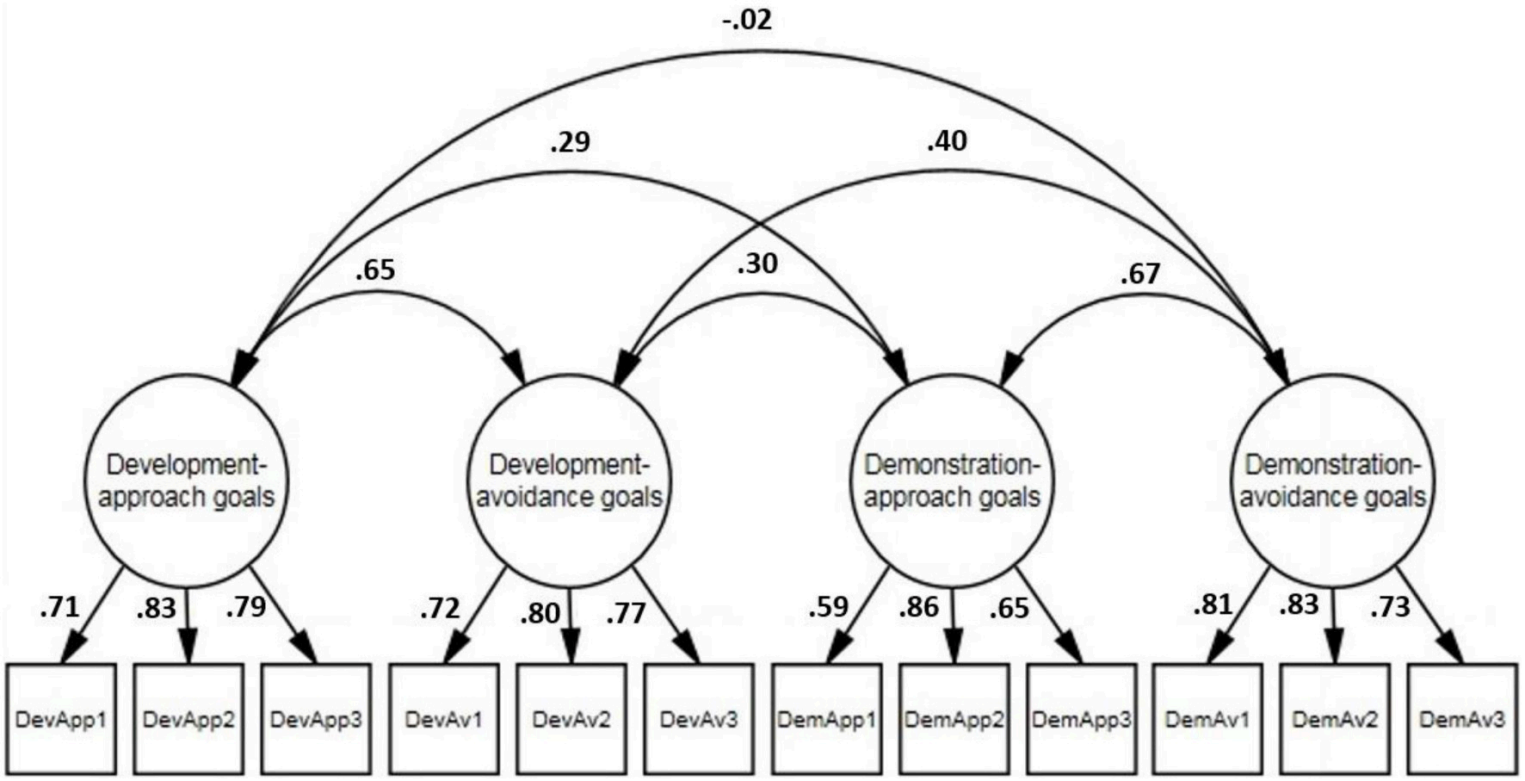
**Confirmatory factor analysis of the 2 × 2 standpoints goal items**. The values in the figure are standardized coefficients.

A confirmatory factor analysis (CFA) was conducted on the achievement goal items using Mplus 7.11 (Muthén and Muthén, 1998–2012). The analysis was conducted on the covariance matrix, and the solution was generated on the basis of maximum-likelihood estimation. Several different indices of fit were used to evaluate the fit of the model to the data. Following the recommendations of Hoyle and Panter (1995), we included both absolute (e.g., chi-square) and incremental [e.g., comparative fit index (CFI)] fit indexes. The CFA examined the hypothesized model, which designated that the items for each goal loaded on their respective latent factors. To identify the model, the variance of each latent factor was fixed to 1 (Bollen, 1989). The results from this analysis supported the hypothesized model, as each fit statistic met the conventional criteria for a good fitting model: χ^2^(48, _*N*__= 244_) = 101.35, *p* < 0.001, CFI = 0.95, TLI = 0.93, RMSEA = 0.07, SRMR = 0.06. Figure 2 presents the factor loadings for this model. Each of the four achievement goals showed adequate internal consistency (Cronbach's α's > 0.76); specific Cronbach's alphas, other descriptive statistics, and intercorrelations are provided in Table 1.

**Table 1:** **Study 1: Intercorrelations and descriptive statistics**.

	**Development-approach goal**	**Development-avoidance goal**	**Demonstration-approach goal**	**Demonstration-avoidance goal**
Development-approach goal	–			
Development-avoidance goal	0.54^**^	–		
Demonstration-approach goal	0.23^**^	0.25^**^	–	
Demonstration-avoidance goal	−0.01	0.34^**^	0.50^**^	–
Mean	3.43	2.67	3.86	3.06
Standard deviation	1.01	1.10	0.79	1.01
Cronbach's α	0.81	0.81	0.76	0.83

** *p < 0.01*.

The fit of the hypothesized 2 × 2 model was compared with five alternative models: (a) an *Undifferentiated* model, in which all items load onto one latent factor; (b) a *Valence* model, in which all similarly valenced items load together onto joint latent factors; (c) a *Definition* model, in which all development-based items load together on a joint latent factor and all demonstration-based items load together on a joint latent factor; (d) a *Trichotomous* model *A*, in which the demonstration-approach and demonstration-avoidance items load together on their hypothesized latent factors, but the development-based items load together on a joint latent factor; (e) a *Trichotomous* model *B*, in which the development-approach and development-avoidance items load together on their hypothesized latent factors, but the demonstration-based items load together on a joint latent factor.

The chi-square difference test, the Akaike information criterion (AIC) and the Bayesian information criterion (BIC) were used to compare the hypothesized 2 × 2 model with the alternative models (see Table 2). The model comparisons indicated that the 2 × 2 model clearly provided a better fit to the data than any of the alternative models.

**Table 2:** **Study 1: Comparison of the hypothesized model and alternative models**.

**Model**	**χ^2^(_*N* = 244_)**	***df***	**CFI**	**TLI**	**RMSEA**	**Δχ^2^(_*N* = 244_)**	**AIC**	**BIC**
2 × 2 Model	101.35	48	0.95	0.93	0.07		7895.85	8041.86
Undiff. model	649.37^**^	54	0.39	0.25	0.22	548.02^**^	8485.90	8611.05
Valence	563.74^**^	53	0.48	0.35	0.20	462.39^**^	8411.05	8539.68
Definition	309.88^**^	53	0.74	0.67	0.14	208.53^**^	8127.75	8256.38
Trichotomous A	240.51^**^	51	0.81	0.75	0.13	139.16^**^	8045.92	8181.50
Trichotomous B	198.67^**^	51	0.95	0.80	0.11	97.32^**^	8002.20	8137.78

CFI, comparative fit index; TLI, Tucker–Lewis index; RMSEA, root-mean-square error of approximation; AIC, Akaike information criterion; BIC, Bayesian information criterion; Undiff, Undifferentiated.

** *p < 0.01*.

Study 1 provided support for the 2 × 2 standpoints model of achievement goals. The data fit the hypothesized model well—better than any plausible alternative—and each of the achievement goal constructs evidenced good internal consistency. Study 2 used this newly designed DAGQ measure to examine the predictive utility of the focal goal constructs for a central outcome in the achievement motivation literature—intrinsic motivation.

## Study 2

### Method

#### Participants and procedure

A total of 405 individuals (243 females, 159 males, 3 unspecified) participated for modest monetary compensation (0.10 USD). The mean age of participants was 34.11 (*SD* = 12.4), with a range of 18–67. Participant ethnicity was as follows: 30 Asian, 40 African-American, 298 Caucasian, 23 Hispanic, 11 “other,” and three unspecified. Participants followed a web link through Amazon's Mechanical Turk to access the study. Participation was restricted to individuals in the U.S. and Canada with an approval rating of 95% or higher who had not participated in Study 1.

A welcome screen communicated that the study focused on individuals' achievement goals. Participants were asked to think about the goals that they have in achievement situations and to choose one domain (school, job, hobbies, etc.) on which to focus. They were then presented with an achievement goal questionnaire to complete with regard to the domain that they had selected. After filling out the questionnaire, participants completed measures assessing intrinsic motivation and social desirability.

#### Measures

Participants' achievement goals were assessed using the DAGQ from Study 1. Participants rated each item on a 1 (*not at all true of me*) to 5 (*extremely true of me*) scale, and their responses were averaged for each goal construct to compute the four achievement goal indexes.

Intrinsic motivation for participants' selected domain was assessed using a general form of Elliot and Church's (1997) eight-item Intrinsic Motivation measure (e.g., “I think this achievement situation is interesting”). Participants rated each item on a 1 (*strongly disagree*) to 7 (*strongly agree*) scale, and their responses were averaged to compute an intrinsic motivation index.

Social desirability was assessed using the 20 self-deceptive enhancement items from the Balanced Inventory of Desirable Responding (Paulhus, 1991). Participants rated each item on a 1 (*strongly disagree*) to 7(*strongly agree*) scale, and their responses were averaged to compute a social desirability index.

### Results

Descriptive statistics and intercorrelations are provided in Table 3. The results from the same type of CFA conducted in Study 1 again supported the hypothesized model, as each fit statistic met the conventional criteria for a good fitting model: χ^2^(48, _*N* = 405_) = 137.20, *p* < 0.001, CFI = 0.93, TLI = 0.91, RMSEA = 0.07, SRMR = 0.05.

**Table 3:** **Study 2: intercorrelations and descriptive statistics**.

	**Intrinsic motivation**	**Development-approach goal**	**Development-avoidance goal**	**Demonstration-approach goal**	**Demonstration-avoidance goal**
Intrinsic motivation	–				
Development-approach goal	0.23^**^	–			
Development-avoidance goal	0.11^*^	0.35^**^	–		
Demonstration-approach goal	0.12^*^	0.51^**^	0.35^**^	–	
Demonstration-avoidance goal	−0.03	0.17^**^	0.60^**^	0.49^**^	–
Sex	−0.19^**^	−0.02	0.00	−0.07	−0.09
Mean	5.47	4.23	3.26	4.04	3.34
Standard deviation	1.04	0.67	1.12	0.74	1.11
Cronbach's α	0.87	0.65	0.81	0.70	0.82

* p < 0.05,

** *p < 0.01*.

Simultaneous multiple regression analyses were conducted to examine the influence of the achievement goals on intrinsic motivation controlling for sex. Together, the five predictors accounted for 10.9% of the variance in intrinsic motivation, *R*^2^ = 0.109, *F*_(5, 396)_ = 9.73, *p* < 0.001. Development-approach goals positively predicted intrinsic motivation, *F*_(1, 400)_ = 8.62, *p* = 0.004, β = 0.17, 95% CI = [0.09, 0.45], as did development-avoidance goals, *F*_1, 400)_ = 6.05, *p* = 0.014, β = 0.16, 95% CI = [0.03, 0.26]. Demonstration-avoidance goals negatively predicted intrinsic motivation, *F*_(1, 400)_ = 9.72, *p* = 0.002, β = −0.21, 95% CI = [-0.32, -0.07]. Sex was associated with intrinsic motivation, *F*_(1, 400)_ = 17.34, *p* < 0.001, β = −0.20, 95% CI = [−0.62, −0.22], indicating that females scored higher on intrinsic motivation than males. No other variables were significant [demonstration-approach, *F*_(1, 400)_ = 1.24, *p* = 0.266, β = 0.07, 95% CI = [−0.07, 0.27]].

To examine the influence of social desirability on the observed results, new variables were created by residualizing self-deceptive enhancement out of each of the achievement goal and intrinsic motivation variables (social desirability and intrinsic motivation were correlated at *r* = 0.26, *p* < 0.001). The same analyses were then re-run with these residualized variables. Together, the five predictors accounted for 8.7% of the variance in intrinsic motivation, *R*^2^ = 0.087, *F*_(5, 396)_ = 7.53, *p* < 0.001. All of the results from the initial analyses held in these ancillary analyses. Specifically, development-approach goals positively predicted intrinsic motivation, *F*_(1, 400)_ = 7.18, *p* = 0.008, β = 0.16, 95% CI = [0.04, 0.27], as did development-avoidance goals, *F*_(1, 400)_ = 4.02, *p* = 0.046, β = 0.13, 95% CI = [0.002, 0.25]. Demonstration-avoidance goals negatively predicted intrinsic motivation, *F*_(1, 400)_ = 5.24, *p* = 0.023, β = −0.16, 95% CI = [−0.29, −0.02]. Sex was associated with intrinsic motivation, *F*_(1, 400)_ = 17.63, *p* < 0.001, β = −0.20, 95% CI = [−0.61, −0.22], indicating that females scored higher on intrinsic motivation than males. No other variables were significant [demonstration-approach, *F*_(1, 400)_ = 0.29, *p* = 0.587, β = 0.04, 95% CI = [−0.09, 0.16]]. See Table 4 for a summary of the findings with and without sex and social desirability included in the analysis.

**Table 4:** **Studies 2 and 3: Beta coefficients and standard errors from simultaneous regression analyses**.

	**Intrinsic motivation**			**Exam performance**		
	**Goals**	**w/Sex**	**w/Sex and social desirability**	**Goals**	**w/Sex**	**w/Sex and SAT score**
Development-approach goal	0.18^**^ (0.09)	0.17^**^ (0.06)	0.16^**^ (0.06)	−0.01 (0.36)	0.00 (0.36)	0.00 (0.08)
Development-avoidance goal	0.14^*^ (0.06)	0.16^*^ (0.06)	0.13^*^ (0.06)	−0.08 (0.33)	−0.09 (0.33)	−0.05 (0.09)
Demonstration-approach goal	0.08 (0.09)	0.07 (0.09)	0.04 (0.06)	0.22^**^ (0.42)	0.22^**^ (0.41)	0.21^**^ (0.07)
Demonstration-avoidance goal	−0.18^**^ (0.06)	−0.21^**^ (0.09)	−0.16^*^ (0.07)	−0.20^*^ (0.33)	−0.19^*^ (0.33)	−0.19^*^ (0.09)
Sex		−0.20^**^ (0.10)	−0.20^**^ (0.10)		−0.11^*^ (1.40)	−0.10^†^ (0.12)

For intrinsic motivation, the first column presents the coefficients without controlling for sex, the second column presents the coefficients controlling for sex, and the third column presents the coefficients controlling for sex and social desirability. For exam performance, the first column presents the coefficients without controlling for sex, the second column presents the coefficients controlling for sex, and the third column presents the coefficients controlling for sex and SAT score.

† p < 0.10,

* p < 0.05,

** *p < 0.01*.

Study 2 provided support for the predictive utility of the goals of the 2 × 2 standpoints model. Development-approach and development-avoidance goals were positive predictors of intrinsic motivation, and demonstration-avoidance goals were negative predictors. Each of these results was found to be robust when controlling for social desirability. Study 3 proceeded to examine the predictive utility of the focal goal constructs for another central outcome in the achievement motivation literature—performance attainment.

## Study 3

### Method

#### Participants

A total of 336 students (218 females, 116 males, 2 unspecified) enrolled in a psychology course at a university in the northeast U.S. participated for extra course credit. The mean age of participants was 19.37 (*SD* = 1.39), with a range of 17–32^2^. Participant ethnicity was as follows: 78 Asian, 23 African-American, 190 Caucasian, 17 Hispanic, 1 Native American, 17 “other,” and 8 unspecified. Participants followed a web link to a designated website to access the study.

The study was part of a broader series of assessments within participants' psychology course. Participants were presented with an achievement goal questionnaire to complete with regard to their upcoming final exam for the course. Performance on the final exam was the focal outcome variable.

#### Measures

Participants' achievement goals for their final exam were assessed using the DAGQ 6 days prior to the exam. Participants rated each item on a 1 (*not at all true of me*) to 5 (*extremely true of me*) scale, and their responses were averaged for each goal construct to compute the four achievement goal indexes.

Performance attainment was assessed via participants' final exam in their psychology course. The exam was administered in the classroom and was comprised of multiple choice, fill-in-the-blank, and short answer questions. Exam scores were obtained from the professor at the end of the course, and could range from 0 to 100.

General ability was assessed using self-reported SAT scores. When ACT but not SAT scores were available, transformation was used; missing data were imputed using the fully conditional specification method in SPSS version 20.

### Results

Descriptive statistics and intercorrelations are provided in Table 5. Simultaneous multiple regression analyses were conducted to examine the influence of the achievement goals on exam performance controlling for sex. Together, the five predictors accounted for 8.0% of the variance in exam performance, *R*^2^ = 0.08, *F*_(5, 327)_ = 5.69, *p* < 0.001. Demonstration-approach goals positively predicted performance on the exam, *F*_(1, 331)_ = 9.23, *p* = 0.003, β = 0.22, 95% CI = [0.44, 2.05]. Demonstration-avoidance goals negatively predicted performance on the exam, *F*_(1, 331)_ = 4.68, *p* = 0.031, β = −0.19, 95% CI = [−1.35, −0.06]. Sex was associated with performance on the exam, *F*_(1, 331)_ = 4.47, *p* = 0.035, β = -0.11, 95% CI = [−5.73, −0.21], indicating that females scored higher than males. No other variables were significant (development-approach, *F*_(1, 331)_ = 0.000, *p* = 0.99, β = 0.00, 95% CI = [−0.71, 0.70], development-avoidance, *F*_(1, 331)_ = −0.09, *p* = 0.288, β = −0.09, 95% CI = [−0.99, 0.29]). See Table 4 for a summary of the findings with and without sex included in the analysis.

**Table 5:** **Study 3: intercorrelations and descriptive statistics**.

	**Exam performance**	**Development-approach goal**	**Development-avoidance goal**	**Demonstration-approach goal**	**Demonstration-avoidance goal**
Exam performance	–				
Development-approach goal	0.06	–			
Development-avoidance goal	−0.14^**^	0.43^**^	–		
Demonstration-approach goal	0.11^*^	0.55^**^	0.35^**^	–	
Demonstration-avoidance goal	−0.16^**^	0.13^*^	0.69^**^	0.42^**^	–
Sex	−0.14^**^	−0.10	−0.05	−0.09	−0.05
Mean	85.68	3.34	2.90	3.82	3.54
Standard deviation	12.60	0.88	1.10	0.74	0.88
Cronbach's α		0.80	0.84	0.71	0.86

* p < 0.05,

** *p < 0.01*.

To examine the influence of general ability on the observed results, new variables were created by residualizing SAT scores out of each of the achievement goal and exam performance variables (SAT scores and exam performance were correlated at *r* = 0.22, *p* < 0.001). The same analyses were then re-run with these residualized variables. Together, the five predictors accounted for 6.6% of the variance in exam performance, *R*^2^ = 0.066, *F*_(5, 330)_ = 4.65, *p* < 0.001. All of the central results from the initial analyses held in these ancillary analyses. Specifically, demonstration-approach goals positively predicted exam performance, *F*_(1, 334)_ = 8.47, *p* = 0.004, β = 0.21, 95% CI = [0.07, 0.36]. Demonstration-avoidance goals negatively predicted exam performance *F*_(1, 334)_ = 4.88, *p* = 0.028, β = −0.19, 95% CI = [−0.35, −0.02]. Sex was marginally significant in its association with exam performance, *F*_(1, 334)_ = 3.55, *p* = 0.065, β = −0.10, 95% CI = [−0.44, 0.01], indicating that females tended to score higher on the exam than males. No other variables were significant or marginally significant [development-approach, *F*_(1, 334)_ = 0.00, *p* = 0.985, β = 0.00, 95% CI = [−0.15, 0.15]; development-avoidance: *F*_(1, 334)_ = 0.33, *p* = 0.572, β = −0.05, 95% CI = [−0.21, 0.12]]. See Table 4 for a summary of the findings with SAT scores included in the analysis.

Study 3 provided further support for the predictive utility of the goals of the 2 × 2 standpoints model. Demonstration-approach goals were positive predictors of performance attainment, and demonstration-avoidance goals were negative predictors. These findings move beyond self-report to document that these achievement goals predict performance on an important test in a real-world achievement context.

## General discussion

In the present research, we proposed and empirically tested a 2 × 2 standpoints model of achievement goals. The first step in putting this model to test was the creation and psychometric validation of an assessment device to measure the four goals of the model. This first step was accomplished in Study 1, as we developed a brief, face-valid Development-Demonstration Achievement Goal Questionnaire (DAGQ) that showed good structural and psychometric properties (i.e., model fit, internal consistency). The next step, accomplished in Studies 2 and 3, was to link the goals in the model to the two central outcomes in the achievement motivation literature: intrinsic motivation and performance attainment. In Study 2, we focused on intrinsic motivation, and showed that development-approach and development-avoidance goals were a positive predictor and demonstration-avoidance goals were a negative predictor of intrinsic motivation. Given that all of the variables in this study were self-reported, we also reanalyzed the data controlling for social desirability and found that all of the results held up to this more rigorous test. In Study 3, we focused on exam performance in a classroom setting, and showed that demonstration-avoidance goals were a negative predictor and demonstration-approach goals were a positive predictor of performance attainment. Thus, Studies 2 and 3 clearly documented the predictive utility of the goals of the 2 × 2 standpoints model for two centrally important achievement-relevant outcomes.

Our main hypotheses were supported by the data. Development-approach goals evidenced a positive but constrained empirical profile in that they were a positive predictor of intrinsic motivation, but were not a significant predictor of exam performance. These goals keep one appetitively focused on one's own trajectory, which affords a positive, challenge-oriented phenomenological experience during task engagement that facilitates enjoyment. However, the process-oriented focus of these goals may not be ideal for boosting performance on one-time evaluative tasks such as class examinations. Demonstration-avoidance goals evidenced an unequivocally negative empirical profile in that they were a negative predictor of both intrinsic motivation and exam performance. The joint focus on both demonstration and incompetence—that is, on demonstrating that one is not incompetent—appears to be a particularly bad combination that exacts both phenomenological and “bottom-line” costs.

Development-avoidance goals were shown to be a positive predictor of intrinsic motivation, but were not a significant predictor of exam performance. Development-avoidance goals may be considered something of a hybrid form of regulation in that they are a composite of one aspect of competence generally thought to be desirable (development) and another aspect of competence generally thought to be undesirable (avoidance). The influence of these goals on achievement-relevant outcomes may depend on which of these two aspects—development or avoidance—is more salient in a given achievement situation. In Study 2 of the present work, participants reported their achievement goals at the broad, domain-general level (e.g., for their work, hobbies) with no immediate task impending, and this relaxed setting may have promoted a primary focus on the development aspect of the goal that had positive implications for intrinsic motivation. In Study 3, on the other hand, participants reporting their achievement goals for a specific, impending task may have been more likely to hone in on the avoidance aspect of the goal, with less positive implications. Another possibility is that the approach-avoidance distinction is less impactful in the context of development relative to the more evaluative context of demonstration, thereby leading to similar empirical profiles for development-approach and development-avoidance goals across achievement contexts. It is important to highlight, however, that the two goals accounted for *independent* variation in intrinsic motivation, so although both were positive predictors of intrinsic motivation, they were positive predictors of separate variance in intrinsic motivation. Thus, while the present research nicely documents the predictive utility of development-avoidance goals, it also points to the need for additional research designed to further examine their influence.

Demonstration-approach goals were not significant predictors of intrinsic motivation but did positively predict exam performance. Like development-avoidance goals, demonstration-approach goals are something of a hybrid form of regulation in that they are a composite of one aspect of competence generally thought to be undesirable (demonstration) and another aspect of competence generally thought to be desirable (approach). Again, the influence of these goals may be dependent on which aspect of the goal is most salient in the achievement situation. However, it is also possible that in at least some instances, the demonstration component of these goals actually spurs on vigorous effort designed to impress others that may, in the short run and for certain types of tasks, benefit performance (Urdan and Mestas, 2006). Some research has found demonstration-approach goals (or their equivalent) to be a negative predictor of achievement (Hulleman et al., 2010; Senko and Tropiano, in press), whereas others have obtained mixed findings (Grant and Dweck, 2003; Warburton and Spray, 2014). Clearly additional research is needed to determine the conditions under which demonstration-approach goals facilitate and debilitate performance attainment. Regardless, even when effective, this type of regulation is likely not experienced as exciting or enjoyable, given the emphasis on self-presentation and validation.

In the DAGQ, the demonstration-based goals focus on showing competence or not showing incompetence, but they do not specify to whom the competence/incompetence might be shown/not shown. Now that the predictive utility of the goals of the 2 × 2 standpoints model has been documented, a more fine grained analysis of different possible referents or “addressees” (Ziegler et al., 2008) is a logical next step. The target of one's demonstration-based strivings may be peers, teachers, parents, coaches, or bosses, and the specific target that is the focus of regulation may influence the phenomenology and efficacy of goal pursuit^3^. For example, it is possible that striving to demonstrate one's competence to a peer may feel less stressful and evaluative than striving to demonstrate one's competence to an authority figure, such as one's teacher or boss. Moreover, when an authority figure is the demonstration referent, the quality of the relationship between the “demonstrator” and the “demonstratee” may serve as an important moderator variable. Striving to demonstrate competence to an authority figure who one views as unconditionally accepting may be fueled by feelings of gratitude or love and promote a host of positive outcomes. In contrast, striving to demonstrate competence to an authority figure who links competence to acceptance (Elliot and Thrash, 2004) or makes global ability attributions (Kamins and Dweck, 1999) may be fueled by feelings of fear or confusion and produce a number of negative outcomes. These considerations highlight the reality of the achievement-affiliation nexus in everyday achievement contexts, a reality that that has only received only a modicum of research attention, particularly relative to its undoubted import (Elliot and Reis, 2003; Assor and Tal, 2012).

Now that empirical support has been obtained for the structural validity and predictive utility of the 2 × 2 standpoints model, an important next step is to integrate this model with other achievement goal frameworks such as the 2 × 2 standards model. One approach might be to assess the goals of the 2 × 2 standpoints and 2 × 2 standards models simultaneously to test their separateness and differential predictive utility. However, methodologically, having the same participants answer 24 achievement goal items with similar approach and avoidance wording is likely to prompt satisficing (Krosnick, 1999) in many respondents. Thus, instead of assessing all of the goals at once, it may be preferable to focus on a subset of the goals in the models in any given study (e.g., just focus on demonstration-based goals and performance-based goals; e.g., Warburton and Spray, 2014; Senko and Tropiano, in press). Conceptually, although standpoints on competence—development and demonstration—and standards of competence—task/self and other—are clearly distinct constructs, in everyday regulation there are some combinations of these constructs that co-occur more often than others (e.g., a demonstration standpoint with an other-based standard, a development standpoint with a task/self-based standard; see Nicholls, 1984; Dweck, 1986). This suggests a second approach to integration—the study of goal complexes (Elliot and Thrash, 2001; Thrash and Elliot, 2001; Senko and Tropiano, in press) in which the reasons for engaging in achievement behavior and the aims that are pursued while engaging in achievement behavior are combined together within a single construct. Thus, for example, people may try to do better than others (an other-approach standard) in order to demonstrate their competence (a demonstration-approach standpoint) and this combination of standard and standpoint could be assessed and examined together as a goal complex. Importantly, conceptualizing standards and standpoints as separate constructs that can also be joined together allows one to account for many different types of self-regulation, including the commonplace goal complex delineated above (an other-approach standard in the service of a demonstration-approach standpoint) and also less common, but still undoubtedly prevalent, goal complexes such as trying to do better than others (an other-approach goal) in order to develop competence (a development-approach standard)^4^.

As the achievement goal approach to achievement motivation has developed, different variants of models have been proposed and tested. Some researchers have expressed concern that these additional models represent a proliferation of frameworks that threatens parsimony and muddies the conceptual waters (Brophy, 2005; Kaplan and Maehr, 2007). We do not think the 2 × 2 standpoints model endangers achievement goal research in this way; on the contrary, we think it is inevitable and important that this model be proposed and tested. It is inevitable, because both individual distinctions—the development-demonstration distinction and the approach-avoidance distinction—are already recognized as core elements of achievement goals in the literature, and the 2 × 2 standpoints model simply integrates them together. It is important because like the prior integration of the task/self-other and approach-avoidance distinctions, the more differentiated model yields additional conceptual rigor and understanding, and provides enhanced predictive utility and interpretational clarity. Parsimony is not the simplest model, but rather the simplest model that fully covers the conceptual space under consideration. Differentiating standpoints from standards and integrating the standpoints with the approach-avoidance distinction is necessary for full coverage of the achievement goal construct.

Based on our findings, a clear recommendation to teachers would be to encourage the adoption of development-approach goals and to discourage the adoption of demonstration-avoidance goals in their students. We do not recommend that teachers encourage demonstration-approach goals or development-avoidance goals, despite their observed benefits in the present work for several reasons. First, additional research is needed to determine the specific contexts in which such goals are beneficial. Second, additional research is needed to determine the full nomological network of these goals, as it is possible that they are also linked to some undesirable outcomes in achievement settings (e.g., demonstration-approach goals may make students more susceptible to selfish or disagreeable behavior more generally in team or interpersonal contexts; see Darnon et al., 2006; Van Yperen and Orehek, 2013, for analogs). Third, even if subsequent research supports the benefits of these goals, a remaining question would be the viability of instantiating these goals in the classroom in a way that is palatable to and supportive of all students. It is possible that these goals only have positive implications when they emerge naturally from students' dispositional tendencies (see Elliot and Moller, 2003).

A limitation of the present research is that we focused exclusively on North American participants. Some achievement motivation research has suggested that individuals in Eastern countries such as Japan, South Korea, and China, are more avoidance-oriented, focus more on the process of improvement than on outcomes *per se*, and desire to fit in rather than stand out (Dekker and Fischer, 2008; Heine, 2008; Elliot et al., 2012; King and McInnerney, 2014). It would be interesting to extend research on the 2 × 2 standpoints model to these countries and to conduct cross-cultural comparisons of the prevalence and implications of adopting these goals in different cultural contexts (for related work using the 2 × 2 standards model, see King, 2015; Miksza et al., 2016; Poondej and Lerdpornkulrat, 2016). Another limitation is that we focused only on the predictive utility of goal pursuit and did not examine antecedents of goal adoption. Promising candidates for future empirical work on antecedents might be implicit theories of ability (Dweck, 2000), public-private self-consciousness (Scheier and Carver, 1985), and approach-avoidance temperament (Elliot and Thrash, 2010). Finally, it is important to acknowledge that our studies examined achievement goals as prospective predictors and, as such, causal relations were not documented. Future work using longitudinal and experimental designs would be welcomed, accordingly.

In closing, the achievement goal approach to achievement motivation has been of interest to researchers and theorists for decades, and the present research proposes and validates an achievement goal model that has been surprisingly overlooked—the 2 × 2 standpoints model. We think that this model is long overdue, as it integrates the essential conceptual distinction proffered in initial achievement goal theorizing (development-demonstration; Nicholls, 1984; Dweck, 1986) with a central conceptual distinction in scientific psychology more generally (approach-avoidance; Elliot, 1999). This integration has been thoroughly explored with regard to the standards of competence and borne much fruit (Elliot, 2005; Hulleman et al., 2010; Van Yperen and Orehek, 2013); we anticipate the same for this integration with regard to the standpoints of competence. More generally, we hope that providing an organizational structure for the achievement goal literature, as well as adding this missing piece to the achievement goal puzzle, will help improve conceptual coherence, and generate new and exciting research as the achievement goal approach moves through its fourth decade.

## Author contributions

RK contributed to study design, managed and organized data collection, analyzed results, wrote paper drafts, edited paper. AE came up with the study idea, worked on study design, edited paper drafts, and significantly revised the final paper.

### Conflict of interest statement

The authors declare that the research was conducted in the absence of any commercial or financial relationships that could be construed as a potential conflict of interest.

## Footnotes

^1^In prior work, Elliot (Elliot, 1999; Elliot and McGregor, 2001) referred to definition as a central component of competence; in this work, definition was explicitly linked to standards of competence evaluation. In the present work, we refer to the focus of competence, instead, in order to communicate that this component of competence is broader than (i.e., includes, but is not limited to) the definition of competence component.

^2^One participant was 17 years old when completing the demographics questionnaire, but provided informed consent after turning 18. This study was part of a larger project on motivation; data from an unrelated part of this larger project have been published in prior work (Elliot et al., 2016, Study 2b; Gocłowska et al., 2015, Study 2; Weidman et al., 2016, Study 2b). None of the findings from the present study have been reported in any prior research.

^3^The self may also be a referent for one's demonstration strivings, in that one may strive to demonstrate to oneself that one is competent (or not incompetent). Importantly, however, one may also use the self as a standard of competence evaluation without striving to demonstrate to the self that one is competent (or not incompetent); this highlights the distinction between standards of and standpoints on competence that is at the core of the present article.

^4^Elliot (2005) characterized the development and demonstration standpoints as reasons and the task/self and other standards as aims, and the examples that we have just given in the text follow this characterization. We think that this characterization is accurate when the standpoints and standards are considered together in functional relation to one another (i.e., as in goal complexes), because in this instance the standards represent proximal goals (aims) operating in the service of the higher order standpoints (reasons). However, when considered alone, standpoints may themselves be characterized as aims or proximal goals that directly predict behavior. It is in this manner that they have been conceptualized and operationalized in the present research. In short, we think it is critical to bear in mind that the reason-aim distinction is based in functional, not absolute designations, and that standpoints, especially, may take on different functions in different conative contexts.
