# Supplementary material for: The 2 × 2 Standpoints Model of Achievement Goals
Source: Front Psychol. 2016 May 19;7:742. doi: 10.3389/fpsyg.2016.00742 (PMC4871878; doi:10.3389/fpsyg.2016.00742)
Supplement: Supplementary file 1 [file DataSheet1.docx]

Appendix A

*Development-Demonstration Achievement Goal Questionnaire Items*

**Development-approach Items**

1. My focus is “To develop my knowledge.”

2. My aim is “To develop ability.”

3. My goal is “To increase competence.”

**Development-avoidance Items**

1. My goal is “To avoid a decrease in ability.”

2. My focus is “To avoid becoming less competent.”

3. My aim is “To avoid losing my knowledge.”

**Demonstration-approach Items**

1. My goal is “To demonstrate ability.”

2. My aim is “To show competence.”

3. My focus is “To demonstrate that I am knowledgeable.”

**Demonstration-avoidance Items**

1. My aim is “To avoid showing incompetence.”

2. My focus is “To avoid demonstrating inability.”

3. My goal is “To avoid demonstrating that I lack knowledge.”
